# A systematic analysis of apple root resistance traits to *Pythium ultimum* infection and the underpinned molecular regulations of defense activation

**DOI:** 10.1038/s41438-020-0286-4

**Published:** 2020-05-01

**Authors:** Yanmin Zhu, Melody Saltzgiver

**Affiliations:** USDA-ARS, Tree Fruit Research Laboratory, Wenatchee, WA 98801 USA

**Keywords:** Biotic, Plant molecular biology

## Abstract

Apple replant disease (ARD), caused by a pathogen complex, significantly impacts apple orchard establishment. The molecular regulation on ARD resistance has not been investigated until recently. A systematic phenotyping effort and a series of transcriptomic analyses were performed to uncover the underpinned molecular mechanism of apple root resistance to *P. ultimum*, a representative member in ARD pathogen complex. Genotype-specific plant survival rates and biomass reduction corresponded with microscopic features of necrosis progression patterns along the infected root. The presence of defined boundaries separating healthy and necrotic sections likely caused delayed necrosis expansion in roots of resistant genotypes compared with swift necrosis progression and profuse hyphae growth along infected roots of susceptible genotypes. Comprehensive datasets from a series of transcriptome analyses generated the first panoramic view of genome-wide transcriptional networks of defense activation between resistant and susceptible apple roots. Earlier and stronger molecular defense activation, such as pathogen perception and hormone signaling, may differentiate resistance from susceptibility in apple root. Delayed and interrupted activation of multiple defense pathways could have led to an inadequate resistance response. Using the panel of apple rootstock germplasm with defined resistant and susceptible phenotypes, selected candidate genes are being investigated by transgenic manipulation including CRISPR/Cas9 tools for their specific roles during apple root defense toward *P. ultimum* infection. Individual apple genes with validated functions regulating root resistance responses can be exploited for developing molecular tools for accurate and efficient incorporation of resistance traits into new apple rootstocks.

## Introduction

Apple (*Malus domestica* Borkh.) is one of the most popular perennial tree fruits in temperate regions around the world^1^. Apple replant disease (ARD) refers to stunted growth or death of newly planted trees at a replant site where apple or closely related tree species have been previously cultivated. The causal agents of ARD consist of a pathogen complex, including necrotrophic soilborne oomycetes (*Phytophthora* and *Pythium*) and fungi (*Ilyonectria* and *Rhizoctonia*)^2,3^. Among them, *Pythium ultimum* is known to be one of the primary members in this pathogen complex, which has been identified in orchard soils worldwide^2,4,5^. Similar to other root diseases, the effective control of ARD is often hampered because of the persistent survival of soilborne pathogens, which form overwintering structures such as oospores, chlamydospores, and sclerotia^6,7^.

The primary control method for ARD has been pre-planting chemical fumigation of orchard soils to eradicate ARD pathogens^8^. In addition to the high cost, the effects of fumigation are short-lived, and soil fumigation is not feasible after orchard establishment^9^. In addition, the use of these broad-spectrum biocides is under increasing regulatory restriction due to their negative environmental impacts and human health concerns. Cultural disease control methods, such as rotation or fallowing, are either impractical or ineffective in managing ARD^6^. Development and deployment of resistant or tolerant rootstocks can offer a cost-effective, ecologically friendly, and durable approach for ARD management; however, conventional breeding for apple root resistance to soilborne pathogens is a long-term and resource-demanding endeavor^10,11^. Genetics-informed breeding, such as the use of predictive DNA markers, can greatly enhance the precision and efficiency for early selection of desired traits^12^. Elucidating the molecular mechanisms of apple root resistance to ARD pathogens is crucial for implementing a genetics-informed breeding strategy for resistant apple rootstocks^13–15^.

Plants are constantly challenged by abiotic and biotic stresses during their lifetime due to their sessile living nature. Facing abiotic stresses, plants have evolved adaptive systems to adjust growth and reproduction according to the predictable and re-occurring changes (e.g., senescence or vernalization)^16,17^. In contrast, biotic stress from pathogen infection or herbivore attack is a largely unpredictable event. Therefore, well-regulated defense activation with proper control over its duration and strength is critical for plant survival^18–20^. Plant root tissues are constantly exposed to the complicated soil microflora including soilborne pathogens. Roots are the very foundation of the entire plant physiology, as exemplified by the myriad of biological functions they perform, including water and nutrient uptake, storage of assimilates, and mechanical anchoring^21,22^. However, investigating the molecular defense responses in plant roots is more challenging because of the lack of visibility, limited accessibility, perturbation to the root system in conducting in vivo experiments, and heterogeneity of development and differentiation processes of the plant root system^23^. Progress has been made on deciphering the molecular mechanisms underpinning the plant immune responses in the last few decades, although most studies were based on foliar pathosystems.

The current understanding of plant molecular defense responses is derived primarily from studies using foliar pathosystems. Specifics and unique aspects of root defense against soilborne pathogens, especially for perennial tree crops like apple, remain largely unclear^24^. Building on a recent phenotyping effort on apple root resistance traits to *P. ultimum* infection, a systemic approach including a series of transcriptome analyses and subsequent functional validation of selected candidate genes, genotype-specific defense activation patterns and their potential contribution to apple root resistance traits began to emerge. A unique trait to apple, as a rosaceae woody crop, is that the reproduction of apple is self-incompatible or outcrossing in nature, and the apple genome has high-level heterozygosity^1,25^. Each seed in a fruit represents a unique genetic identity, and therefore seed germination cannot generate plants with identical genetic background. This review primarily focuses on the pathosystem between apple roots and soilborne necrotrophic *P. ultimum*, except some additional works on the molecular interactions between apple root and *Rhizoctonia solani* AG-5, another ARD pathogen. This review can be considered as a companion to an earlier perspective review on investigating the molecular basis of apple root resistance to ARD^7^.

## Molecular defense responses in model pathosystems

Plants are equipped with a versatile and tightly regulated immune system through coevolution with their pathogens. Such a sensitive immune system allows plants to discriminate between beneficial and pathogenic microbes in their surroundings^26,27^. Plants use a sophisticated surveillance system to detect the presence of pathogens and subsequently initiate appropriate defense responses according to the pathogen types^19,28^. It has been demonstrated that pattern recognition receptors (PRRs) located on the cell membrane detect the signature molecules or pathogen-associated molecular patterns (PAMPs), which are often conserved within the same class of pathogens^29^.

An optimized defense output requires coordinated reprogramming of cellular processes and efficient redirection of metabolic activities in plant cells. Plants utilize a two-layer immune system, or zig-zag model, to combat pathogen aggression^20,28^. Upon PRRs perceiving the conserved PAMP, plants activate a process known as PAMP-triggered immunity (PTI)^30,31^, which adapted pathogens can suppress or bypass by secreting evolved effector proteins^20,32^. On the plant side, co-evolved resistance (R) proteins directly or indirectly interact with pathogen-derived effectors and initiate the second layer of defense, i.e., effector-triggered immunity (ETI)^20,32,33^. PTI is generally considered to be a basal immune reaction, and ETI can lead to a stronger and more specific defense response toward those pathogen isolates that produce the recognized effector.

It has been well established that plant hormones, such as salicylic acid (SA), ethylene (ET), and jasmonic acid (JA), are the vital components of plant defense responses^34–37^, and plants use discrete hormone balances and fine-tuning of crosstalk to deal with various attackers. SA-regulated defense mechanisms are activated in response to biotrophic pathogens, whereas JA/ET-mediated signaling pathways are critical to plant defense responses to necrotrophic pathogens^34,38,39^. Crosstalk with other plant hormones, such as auxin, abscisic acid (ABA), and gibberellic acid (GA) can result in multiple feedback loops to modulate gene expression patterns and feed-forward loops to coordinate expression intensity and duration of specific genes^40,41^. Responding to specific hormones or other defense signals, several families of plant TFs are activated, including WRKY (containing WRKYQK protein domain), ERF (ethylene response factor), and MYB (myeloblastosis oncogene)^42,43^.

Both preformed antimicrobial compounds (phytoanticipins) and pathogen infection-induced production of antimicrobial secondary metabolites (phytoalexins) are believed to contribute to pathogen resistance^44–46^. Phytoalexins are small molecules of extreme structural diversity, and they can be generally categorized into three main classes of phytochemicals, including terpenoids, phenylpropanoids, and alkaloids^47,48^. More recently, cellular small RNAs (sRNAs), including microRNA (miRNA) and small-interfering RNAs (siRNA), have been demonstrated to be actively participating in both host immunity and pathogen virulence^49^. In many cases, miRNAs are known to regulate PTI via targeted transcripts of genes functioning in hormonal signaling or as TFs. At the same time, numerous miRNAs have been shown to directly target transcripts of NB-LRR (nucleotide binding/leucine-rich repeat) genes, a class of resistance (*R*) genes predominantly functioning in ETI^50,51^.

## Specific obstacles for unraveling the molecular defense mechanisms in apple roots to soilborne necrotrophic pathogens

Molecular regulation of defense activation in apple roots upon infection from soilborne necrotrophic pathogens, such as those inciting ARD, has not been investigated until recently^7,52–56^. Previously observed ARD resistance or tolerance for selected apple rootstocks based on field evaluation likely involve a combination of multiple functional mechanisms, such as fine root development^57,58^. Well-designed experiments under controlled conditions are required to minimize compounding factors, as reliable and detailed apple root resistance responses are the prerequisite for analyzing the underlined molecular regulation of apple root resistance to *P. ultimum* infection^13^. However, compared with the aerial parts, phenotyping resistance traits of plant roots is more challenging. The hidden nature of root systems in soil limits accessibility and hinders the noninvasive, nondestructive evaluation of their detailed resistance responses, though recently developed methods allowed direct observation of apple root growth behavior in a pot with a transparent wall^59^. In addition, the small stature of the individual young (feeder) roots presents a challenge for direct observation and documentation of the detailed features of apple root pathogenesis processes^24,60^. The heterogeneous or non-synchronized differentiation processes among individual root branches present another practical hurdle for consistent evaluation of apple root resistance behaviors. More significantly, an extra obstacle for apple as a rosaceous species is the continued availability of plant root tissues for repeated infection assays.

Many horticultural traits and disease tolerance of apple rootstocks were traditionally evaluated under field conditions using stool-bed propagated 1-year-old rootstock “sticks” or trees from commercial nurseries^10,61,62^, although using of in vitro-propagated apple plants is becoming more common^63–65^. For example, a comprehensive screening of ARD tolerance was carried out among multiple apple rootstock genotypes using in vitro propagation and steam-disinfected ARD soil as control, and under greenhouse conditions^65^. In most cases, physiological parameters, including tree height, stem diameter, and accumulated fruit yield, were used to infer tolerance to ARD indirectly, without inquiring the intrinsic pathological features in apple roots under pathogenic pressure. Field performance of a rootstock can be influenced by multiple factors, including root regeneration dynamics, nutrition uptaking efficiency, adaptability to certain soil types, and the effect of scion genotypes on rootstock activities^10^. While it is a viable approach to assess the overall rootstock performance to disease pressure under field conditions, it can be problematic using these nursery-generated rootstock trees to collect consistent and detailed resistance phenotypes. Moreover, the availability of these 1-year-old bare-root trees is generally restricted to a few elite commercial varieties, as well as being limited to a short time window during the year. In addition, the root systems of these trees have been exposed to various soil microbes or impacted by inadvertent abiotic conditions in the nursery. More importantly, due to the destructive nature of pathogen infection, the reliability of phenotype data requires repeated infection assays. Therefore, a continuous supply of plants from any apple rootstock genotype is crucial for a detailed and systematic phenotyping effort. Plant tissue culture based on in vitro micropropagation of apple plants is the method of choice for this purpose.

**Fig. 1:**
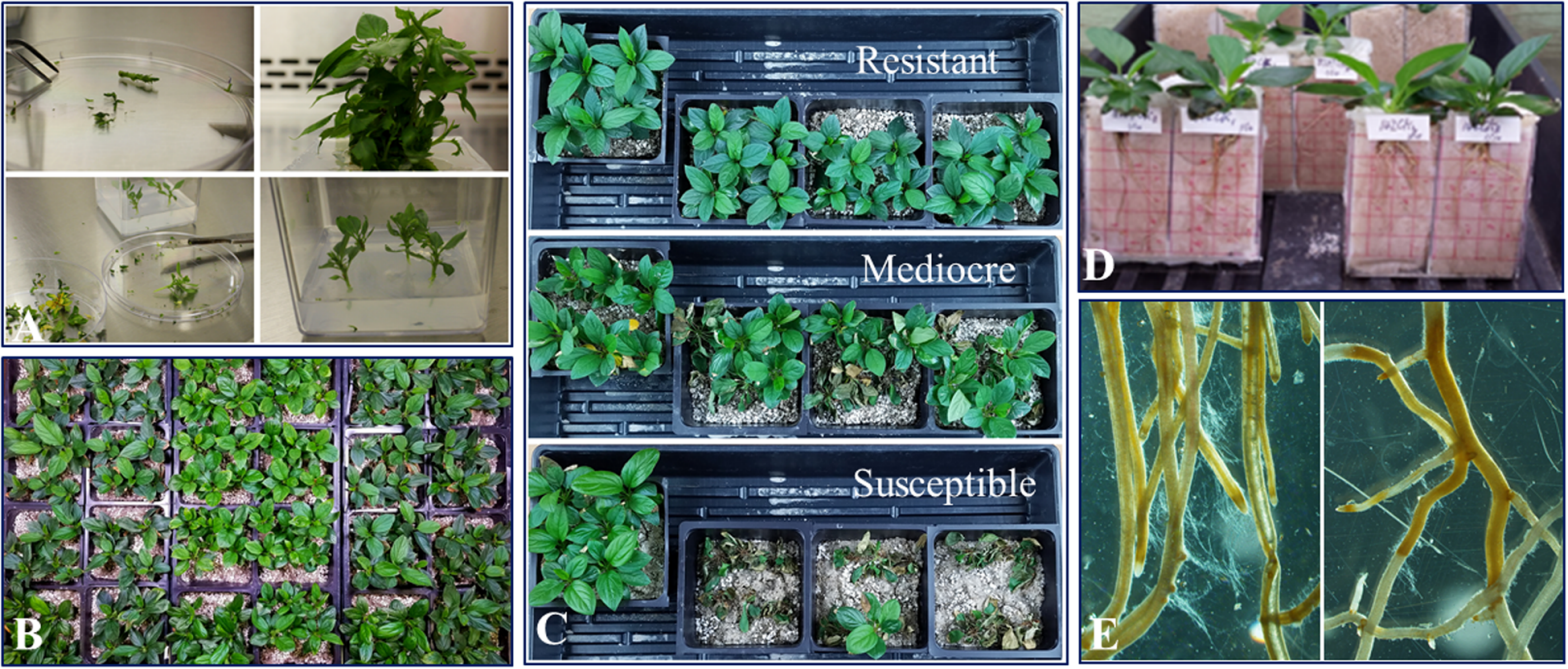
Illustrated processes for phenotyping apple root resistance responses. **a** The simplified steps related to in vitro micropropagation of apple plants by tissue culture procedure, clockwise from top left: shoot meristem for shoot proliferation; proliferated shoot tips; processed shoot tips for root induction; shoot tips for root induction before root elongation. **b** Uniform young apple plants with comparable size and age for selected genotypes were generated by a synchronized micropropagation procedure for simultaneous infection assays. **c** Representative images exhibiting variable and repeatable survival rates at 7 dpi, among resistant, mediocre, and susceptible genotypes (from top to bottom) in the same infection assay and using the identical inoculum preparation; the plants in pots at the left end of each row were the respective mock inoculation controls. **d** Custom-made glass pots used for non-interruptive and nondestructive observation of root pathogenesis process under a dissection microscope. **e** Representative necrosis progression patterns observed under a dissection microscope. The left panel for a typical susceptible genotype: widespread necrotic tissues with the semitransparent appearance, yellow-brownish coloration, and profuse growth of pathogen hyphae at 48 hpi; right panel for a resistant genotype showing limited necrosis as indicated by the presence of clear-cut “boundaries” separating healthy (white and intact) and necrotic sections of roots, minimal hyphae if visible

Plant tissue culture, as a century-old technique and based on the concept of totipotency, represents an unparalleled methodology to propagate clean and healthy plants^66^. The synchronized micropropagation procedure allowed the simultaneous analysis of the root resistance responses for multiple genotypes to the infection by the same pathogen inoculum preparation (Fig. 1a, b)^67^. The small size of 4-week-old apple plants offers the advantage of easy handling under lab and greenhouse settings. While setting up the phenotyping protocol, it was determined that a period of 1-week in-soil acclimation is required for the root system to consistently express the inherent resistance traits^68,69^. In summary, the implementation of an apple micropropagation procedure, accompanied by standardized inoculation methods and a variety of phenotyping methods, sets the foundation for systematic, detailed, and reliable evaluation on apple root resistance traits.

## Systematic characterization of root resistance phenotypes to infection by *P. ultimum* among apple rootstock germplasm

Oomycete pathogen *P. ultimum* is one of the primary components in ARD pathogen complex in Washington State and other regions around the world^3^. Although many chemical, physical, and biological factors are known to contribute to ARD incidence, the primary reason for selecting *P. ultimum* as a representative ARD pathogen is the feasibility to quantify the inoculum level by oospore count^70,71^. Use of quantified inoculum levels is important for repeatable and consistent resistance evaluation between infection events and/or among rootstock genotypes^68,69^. The phenotyping protocol consists of three integrated modules: (1) Continuous supply of comparable apple plants for selected rootstock genotypes by tissue culture-based micropropagation; (2) quantifiable inoculum level and standardized inoculation protocol for consistent resistance evaluation; (3) multiple methodologies developed for evaluating root resistance responses at whole-plant and tissue levels. The phenotyping protocol was initially established using two apple rootstock genotypes, G.935 and B.9, with reported ARD tolerance and susceptibility under field conditions, respectively^62^. Subsequently, a systematic evaluation of resistance responses was carried out for more than 60 F1 progeny derived from a cross between “Ottawa 3” × “Robusta 5” (O3R5), two elite apple rootstock parents^53^.

A wide spectrum of resistance responses was observed among the tested O3R5 genotypes (Fig. 1c). The genotype-specific plant survival rates ranged from single digits to over 90%^53^. This observation indicated that the level of pathogen inoculum (2 × 10^3^ oospores) and root dipping as the inoculation method can effectively distinguish between the resistance levels among tested germplasm^68^. The whole-plant resistance responses, including partial wilt or plant mortality, were visually evaluated daily and recorded at 3, 7, 10, 14, 21, and 28 dpi (days post inoculation). Although the overall survival rate was assigned based on the data at 28 dpi, genotype-specific plant mortality was generally stabilized at 7 dpi. Some genotypes with extreme susceptibility exhibited observable wilting symptoms as early as 3 dpi^53,68^. For simplicity, those genotypes showing consistent survival rates of less than 30% were designated as “susceptible”, and those greater than 80% as “resistant”^53^. Biomass reduction (for either root or shoot) showed statistically significant differences at 28 dpi, when the values of the surviving plants from *P. ultimum* inoculation were compared with those of mock-inoculated control plants for most susceptible genotypes. The opposite held true for the more resistant genotypes^53^. Selected genotypes with distinct resistance responses were also subjected to a more focused microscopic examination.

Microscopic observation of infected root tissues revealed several features that were associated with whole-plant resistance responses^53,68,69^. First, using a custom-made glass-box pot (Fig. 1d), the partially exposed apple root system provided a method for continuous observation of symptom development under a dissection microscope. The time-lapsed images indicated a contrasting pattern of necrosis progression^53^. A swift development of root necrosis was observed for the most susceptible genotypes, with the entire root system becoming necrotic within a period of 24 h after initial infection was identified (Fig. 1e, left panel). For the more resistant genotypes, the necrosis progression could be delayed for several days without the entire root tissues being engulfed^53^. Second, the presence of a well-defined boundary separating healthy and necrotic root sections was frequently identified along the infected roots of resistant genotypes (Fig. 1e, right panel). A similar phenomenon was rare, if present at all, for the more susceptible genotypes^53,69^. Third, profuse growth of *P. ultimum* hyphae was frequently associated with infected roots of susceptible genotypes, but not resistant genotypes (Fig. 1e). The delayed necrosis progression along the roots of the resistant genotypes, such as O3R5-161, suggested the existence of an effective defense mechanism for reducing pathogen development. In contrast, the swift expansion of necrotic tissues accompanied with profuse pathogen hyphae growth along the root section of the susceptible genotypes, such as O3R5-132, clearly demonstrated an inability to restrict the pathogen progression^53^. In summary, a panel of germplasm with contrasting and repeatable resistance responses were identified from this systematic phenotyping effort. These plant materials are valuable for subsequent functional genomic studies to unravel the molecular networks regulating resistance and susceptibility in apple roots to *P. ultimum* infection. The developed methodology, particularly the use of a small glass-box pot and microscope-assisted examination, provided the never-before described methodology for documentation of apple root resistance responses based on continuous, nondestructive observations. Given the challenging nature of phenotyping root interaction with soilborne pathogens, this established phenotyping method represents a significant advancement for investigating apple root resistance traits with improved consistency and repeatability^68,69^.

## Omics approaches to identifying genome-wide networks and specific candidate genes regulating apple root resistance

The timing, intensity, and dynamics of plant defense responses vary depending on the pathosystem, plant genotypes, and tissue types. Until recently, knowledge was essentially nonexistent regarding the molecular regulation of apple root defense activation under pathogenic pressure^52,54–56,63,64,69^. Transcriptional regulation is a fundamental aspect of gene function over a biological process. Therefore, transcriptome analysis represents the most accessible option for a less-defined biology, such as resistance responses of apple roots to ARD pathogens. With its enormous capacity and high-fidelity representation of each activated gene, RNA-sequencing (RNA-seq)-based transcriptome analysis can simultaneously identify and quantify the entire inventory of the expressed genome in apple root tissues. Improved bioinformatic software can offer a wide-angled and high-resolution view of transcriptome landscapes in apple roots during interaction with *P. ultimum*^72^.

**Fig. 2:**
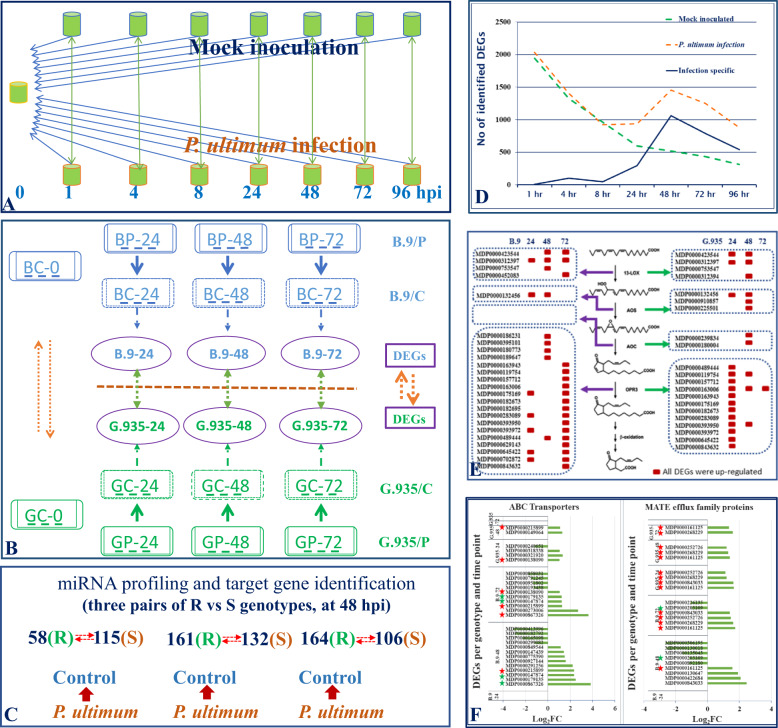
Consecutive transcriptome analyses to identify the genome-wide transcriptional changes specifically associated with apple root defense activation to *Pythium ultimum* infection. **a** Transcriptome survey for comparisons between treatments among eight time points. **b** Comparative transcriptome profiling to identify the differential transcriptional regulations in response to infection by *P. ultimum* between a resistant (G.935) and a susceptible (B.9) genotype. **c** Focused miRNA profiling to identify target genes using three pairs of resistant and susceptible genotypes at the critical stage of 48 hpi. **d** A result displaying the time course of molecular defense activation in apple root in response to infection by *P. ultimum*. **e** Genes in the JA biosynthesis pathway illustrating the early and strong activation in the roots of the resistant G.935 genotype, as a direct comparison to the delayed and interrupted induction in the root of the susceptible B.9 genotype. **f** DEGs encoding two families of transporters showing differential regulation patterns between resistant and susceptible genotypes, early and consistent upregulation in the resistant genotype G.935, in comparison with delayed and partial downregulation in the susceptible genotype B.9

Using *P. ultimum* as a representative ARD pathogen, the primary goal of the first (out of a series of three) transcriptome analysis was to reveal the timeframe of molecular defense activation, and to identify the main categories of differentially expressed genes (DEGs) in infected apple roots^56^. By comparing the transcriptomes of mock-inoculated and *P. ultimum*-inoculated roots, the results clearly demonstrated that the apple root defense response peaked at 48 hpi (hour post inoculation) among eight time points ranging from 0 to 96 hpi (Fig. 2a, d). Using twofold change as the cutoff value, about 2000 DEGs were identified at 48 hpi, after which the intensity of transcriptome changes receded from 72 to 96 hpi^56^. Genes encoding proteins with predicted functions in the pathway of pathogen detection, such as receptor-like kinases (RLKs) and wall-associated receptor kinase (WAKs), were among the notable groups of regulated apple genes. Genes encoding proteins functioning in the biosynthesis and signaling of several plant hormones, including JA, ET, and cytokinin, made up the most recognizable functional groups. Genes encoding enzymes for secondary metabolisms, cell wall fortification, and pathogenesis-related (PR) proteins, laccase, mandelonitrile lyase, and cyanogenic beta-glucosidase represented the wide spectrum of cellular activities with the effort to ward off pathogen progression^56^. The results from this dataset, particularly the timeframe of defense activation, served as a valuable guideline for designing subsequent experiments to compare transcriptome changes between a resistant and a susceptible apple rootstock genotype^52,55,69^.

A subsequent comparative transcriptome analysis was designed to detect the genotype-specific patterns of defense activation in the roots between the susceptible genotype B.9 and the resistant genotype G.935 in response to *P. ultimum* infection. About half a billion paired-end 150-bp reads were generated using the Illumina Solexa HiSeq 3000 platform, with the experimental design encompassing two treatments (mock-inoculated and *P. ultimum* inoculated), three biological replicates, and four time points (0, 24, 48, and 72 hpi) for each genotype (Fig. 2b). Side-by-side comparison of the identified DEGs from each genotype revealed a panoramic view of transcriptome changes with contrasting patterns of multiple defense-related pathways between resistant and susceptible genotypes^52^. One of the most notable features were the overrepresented DEGs with downregulated patterns from susceptible B.9, indicating a widespread suppression of multiple cellular processes. DEGs with annotated functions, such as kinase receptors, MAPK signaling, JA biosynthesis enzymes (Fig. 2e), TFs, and transporters, appeared to be readily induced early at 24 hpi and continued their upregulation at 48 hpi in the root of resistant G.935. In sharp contrast, delayed and/or interrupted activation of multiple defense pathways seemed to be specifically associated with the susceptible B.9 (Fig. 2e, f)^52^. Lack of weakened ETI or existence of a susceptibility gene (such as two induced MLO homologous genes) were speculated for this severely disturbed transcriptome and the resulting susceptibility in B.9 roots^52^. The results also revealed that, even before the presence of pathogen, a preformed molecular defense network appeared to be robustly functional in the roots of the resistant G.935, but to a lesser degree in B.9^55^. Although the existence of a constitutively expressed molecular defense network seems to be contradictory to the theory of trade-off between growth and defense, enhanced readiness for an organ (like root) may also be evolutionarily beneficial, as it is more than likely to encounter adverse biotic stress^73^. These RNA-seq datasets offered the first comprehensive view of molecular defense activation to *P. ultimum* infection and identified a list of candidate apple genes that may potentially play a role in differentiating resistance from susceptibility of apple roots. In addition, by taking advantage of the huge dataset of gene expressions in apple roots, a set of stably expressed apple genes were validated as the preferred reference genes, which are valuable for subsequent functional validation using qRT-PCR technique^74^.

**Fig. 3:**
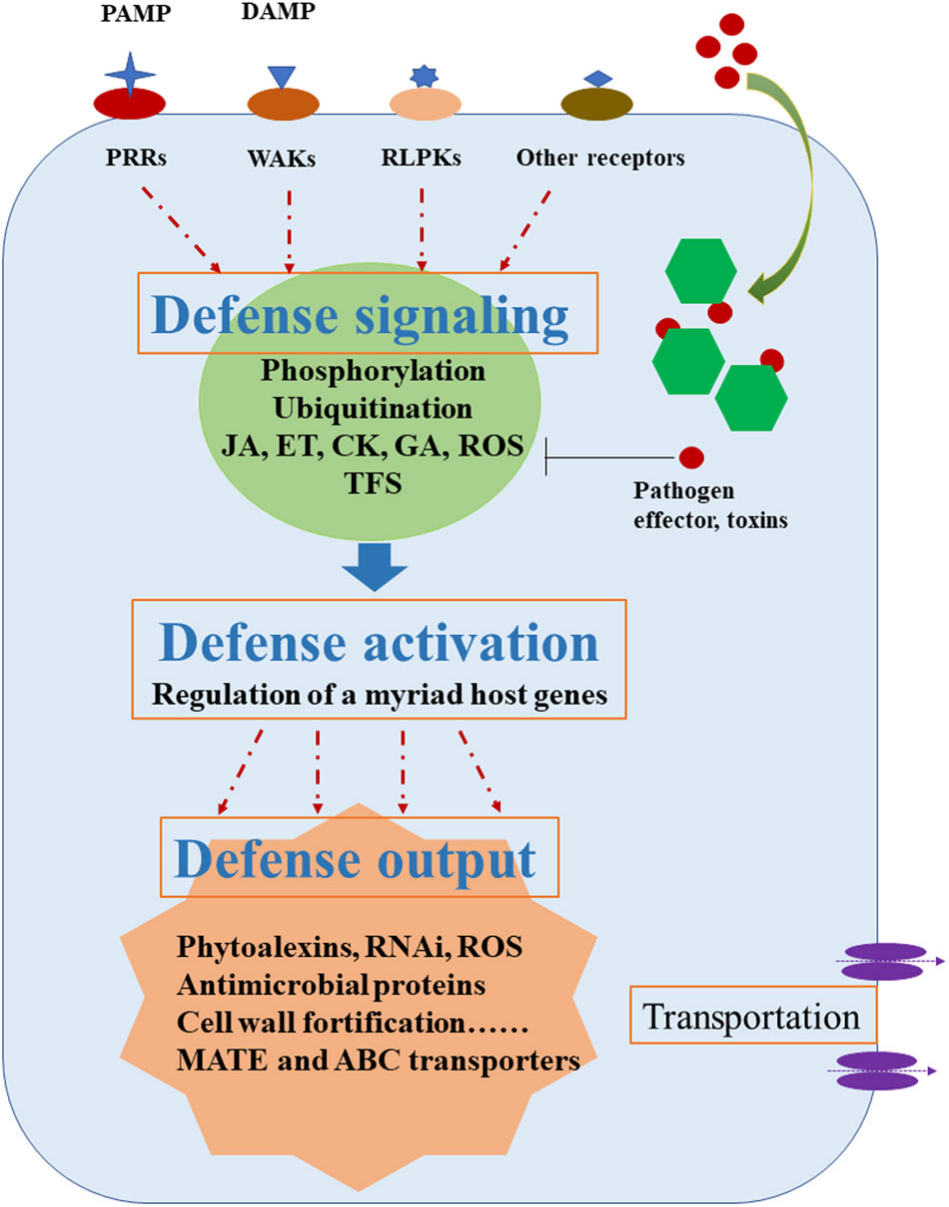
Illustrated molecular defense responses in apple roots under pathogenic pressure from *Pythium ultimum*. An elaborate surveillance system including cytoplasm membrane-localized receptors and receptor kinases (oval shape with various colors) can detect the presence of pathogen by recognizing PAMPs and DAMPs (stars, triangle, and diamond in blue color), which initiate the cascade of defense signaling such as phosphorylation or ubiquitination of cellular proteins. The fine-tuned defense signaling leads to defense activation, including phytohormone biosynthesis and/or ROS generation, as well as induction or repression of TFs. As a result of defense activation, extensive transcriptional reprogramming leads to multifaceted and specific defense outputs, including the production of antimicrobial compounds and pathogenesis-related proteins. Multiple transporters may play critical roles in delivering these antimicrobial components to infection sites for neutralizing and restricting pathogen aggressiveness. The effectiveness of these cellular processes, including the duration, intensity, and temporal/spatial expression patterns of defense genes, may dictate the outcome of the interactions between plant and pathogen, and lead to either a cellular collapse of host cells and plant mortality, or effective inhibition of pathogen progression survival of infected plants. PAMP pathogen-associated molecular pattern, DAMP damage-associated molecular pattern, PRR pattern recognition receptor, WAK wall-associated kinase, RLPK receptor-like protein kinase, ROS reactive oxygen species, JA jasmonic acid, ET ethylene, CK cytokinin, GA gibberellic acid, MATE multidrug and toxic compound extrusion, ABC transporter ATP-binding cassette transporter, TF transcription factor

The regulatory role of sRNA has been unequivocally demonstrated recently in silencing selected targeted genes related to plant immune responses^51,75,76^. The preferential targets include those genes encoding R proteins, transcription factors (TFs), hormone biosynthesis and signaling^51^. Understanding the regulatory roles of sRNAs and identifying their target genes is crucial for further pinpointing the major cellular processes and key candidate genes regulating apple root resistance. Using a selected panel consisting of three pairs of susceptible and resistant apple rootstock germplasm, a focused miRNA profiling analysis and the associated degradome analysis are being carried out to identify the miRNA families and their target genes at the key stage of pathogenesis at 48 hpi (Fig. 3c). These results should provide a unique perspective for elucidating the potential key regulators or pathways controlling apple root resistance to infection by *P. ultimum*. Library sequencing is complete, and the data analysis is currently underway. Cell membrane-located plant PRRs have been well established as playing central roles in triggering defense responses by binding the highly conserved PAMPs^29^. The so-called PTI represents the first layer of defense activation^20,28,36^. Based on transcriptional profiling and proteomic analysis, apple gene MD09G1111800 was shown to encode *MdCERK1* (chitin elicitor receptor kinase 1), a functional PRR during apple root–*Rhizoctonia solani* (AG-5) interaction^54^. *MdCERK1* was expressed primarily in the vegetative tissue of root and leaf, and its expression levels in apple root were induced in response to chitin treatment. The ability of purified GST-MdCERK1 fusion protein to bind chitin molecules added biochemical evidence to its role in chitin-mediated immune responses. An untargeted proteomic approach identified its putative in vivo- interacting partners, including PR-4 protein in apple roots inoculated with *R. solani*. These data support the conclusion that MdCERK1 is a chitin-binding receptor kinase that functions in apple root defense activation. Transgenic manipulation on its in planta expression is underway for validating its genetic identity in apple root resistance to *R. solani*. These omics-based analyses offered a wide-angle view of molecular defense responses and identified a list of apple candidate genes. The subsequent functional analyses should determine their potential contribution to apple root resistance to soilborne necrotrophic ARD pathogens.

## Perspective on functional validation of selected candidate genes for their roles in apple root resistance to *P. ultimum*

Based on the previous transcriptome analyses^52,55,56^, several groups of candidate genes were selected for further investigation. These include candidate genes functioning as receptors, TFs, hormone signaling, R proteins, and secondary metabolism pathways. The consistent transcriptional profiles among expanded groups of apple rootstock germplasm should be the preliminary evidence for their association with resistance traits. The more definitive evidence of their molecular functions in contributing to resistance/susceptibility can be derived from the in planta manipulation of their expression by gene knockout or overexpression. Candidate genes with robust correlations between gene expression patterns and resistance levels are being further investigated using transgenic manipulation. The transgenic approach is particularly valuable for a non-model plant like apple as a perennial tree crop, which has a high level of heterozygosity in its genome and lacks the feasibility of applying large-scale and high-throughput mutagenesis^1,25,77^. *Agrobacterium*-mediated genetic transformation as a delivering system for introducing recombinant DNA (or transgene) has been a well-developed tool for basic research and crop improvement for several decades^78^. More recently, CRISPR/Cas9 (clustered regularly interspaced short palindromic repeats and CRISPR-associated protein 9) technology by double-strand break-mediated genome editing has gained incredible momentum in recent years. For its accuracy on site-directed mutagenesis and applicability to a wide range of organisms, the CRISPR/Cas system has emerged as the most promising tool for targeted mutagenesis^79–81^. Taking the advantage of our existing tissue culture platform, transgenic lines with knocked-out target genes have been generated. Analyzing the potential phenotypic alterations will provide critical insights for assigning the functional identity of tested candidate genes during apple root defense response to *P. ultimum* infection.

Many biochemical assays for detecting early and late cellular defense responses have been reported, such as ROS burst, callose deposition, and lignin formation, as well as enzymes catalyzing the generation of antimicrobial phytoalexins^82–84^. For example, deposition of callose, a high-molecular-weight β-(1, 3)-d-glucan polymer, is a common form of plant defense response for cell wall fortification at the infection site^84,85^. Besides functioning as a physical barrier to pathogen progression, callose at the nanopore structure in cell walls is believed to also serve as a platform for the directed deposition of antimicrobial compounds^84,85^. Lignification is another commonly observed cell wall reinforcement for restricting pathogen advances. Cinnamyl alcohol dehydrogenase (CAD) is a major biosynthetic enzyme, which catalyzes the oxidative cross-linking of the monolignans into long-chain polymers of insoluble lignin^86^. Our transcriptome analyses have identified multiple apple callose synthase and CAD-encoding genes that are specifically downregulated in the root of the susceptible B.9 genotype at 48 hpi^52^. Methodologies of histochemical staining and image-based quantification^87–89^ will validate the relationship between the genotype-specific patterns of cell wall enforcement and observed apple root resistance traits. Several assays were reported for determining the enzymatic activities related to hormone biosynthesis and secondary metabolism, such as lipoxygenase (LOX), chalcone synthase (CHS), and phenylalanine ammonium lyase (PAL)^82,90^. These biochemical analyses and enzymatic assays will be valuable tools to add evidence for validating the functional identity, and to assess their contribution to apple root resistance or susceptibility.

## Concluding remarks and prospects

Maximized exploitation of plant genetics for managing root diseases requires dissecting the molecular regulation networks controlling plant root defense activation and resistance traits toward soilborne pathogens. In the post-genomics era, a lack of high-quality phenotypic data remains a major operational bottleneck for genetic studies on target traits, and therefore hinders the realization of genetic potential contributing to agricultural productivity and sustainability^13,91^. For a perennial tree crop like apple, multiple obstacles stand before the systematic and reliable characterization of the detailed root resistance traits. The lack of a continuous supply of genetically uniform apple plants for repeated infection assays and limited accessibility for detailed characterization of root pathogenesis processes are two examples. Although it is a laborious and time-consuming procedure, in vitro micropropagation of apple plants allowed consistent and detailed phenotypic analysis for more than 60 apple rootstock genotypes. In addition, using custom-made glass-box pots, along with the assistance of a dissecting microscope, allowed the non-interruptive and nondestructive observation of symptom development on the partially exposed apple roots^53,68^. For the first time, genotype-specific resistance traits of apple roots to *P. ultimum* infection were described in detail at both whole-plant and tissue levels. Along the infected roots of resistant genotypes, the presence of the defined boundaries separating healthy and necrotic sections likely resulted in the limited necrosis expansion and indicated an effective resistance mechanism. In contrast, the swift necrosis progression and the profuse growth of pathogen hyphae most likely indicated the lack of an effective defense and failed effort to restrict pathogen development^53^. The phenotyping effort using these innovative methodologies resulted in a panel of apple rootstock germplasm with well-defined resistance levels. The availability of this set of apple rootstock germplasm provides a solid foundation for meaningful molecular analysis aimed to understand resistance mechanisms and subsequent exploitation of natural resistance in the future.

The comprehensive transcriptome datasets from a series of experiments offered the first panoramic view of genome-wide transcriptional networks regulating apple root defense activation toward *P. ultimum* infection^52,55,56^. As illustrated in Fig. 3, the results suggest that successful defense activation consists of several coordinated processes in infected apple roots. The earlier and stronger defense activation, such as pathogen perception and hormone particular JA signaling, likely functions as key differentiating points in conferring apple root resistance^52^. Delayed and interrupted defense activation, such as those related to production and transportation of antimicrobial secondary metabolites, may have resulted in insufficient defense. The inability to effectively cope with pathogen toxins, weakened ETI, or existence of a susceptibility gene are other possible factors to a highly disturbed transcriptome in the root of the susceptible genotype. Using the panel of apple rootstock germplasm with defined resistance traits from the recent phenotyping efforts, experiments are being carried out with the aim to establish the correlative or causal relationship between individual candidate genes and root resistance traits. The functional identities of selected apple candidate genes are being validated through investigation of their induction patterns among an expanded apple rootstock germplasm set, transgenic manipulation, and subsequent biochemical analyses on potentially altered resistance phenotypes.

Multiple genetic and/or environmental factors contribute to the molecular controls of apple root resistance traits. Therefore, many questions related to resistance in apple roots remain unanswered and will need to be addressed with future studies. For example, will the observed resistance traits be the same in the older plants? Do the observed resistance phenotypes to a singular pathogen and under controlled conditions bear any similarity with the overall apple rootstock resistance to ARD under field conditions? What are the effects of scion cultivars on rootstock resistance? Perhaps more relevant is that under field conditions, plant roots interact with a plethora of nonpathogenic and symbiotic microorganisms in addition to pathogens^24^; therefore, current understanding of apple root resistance responses needs to be further scrutinized in the context of apple root living in a complex soil microbiome. Answers to these questions are beyond the scope of the current study, and novel approaches and continuing investigations are certainly required. The current review attempts to summarize the progress on the systematic phenotyping effort on apple root resistance traits and transcriptome analyses on genome-wide activation of defense responses in response to *P. ultimum* infection. The developed phenotyping methodology and resulted plant materials with defined resistance traits, as well as acquired information on genotype-specific molecular defense mechanisms, represent a significant advancement for this minimally investigated research topic. Recently, substantial progress has been made on the investigation on the molecular regulation of apple root resistance/tolerance to ARD, using a reference apple rootstock genotype “M26” and ARD soil^63,64,92^. Integration of current knowledge and additional analytic capability from other branches of omics will be necessary, such as metabolomic analysis for identifying specifically enriched antimicrobial compounds during *P. ultimum*-root interaction. The better understanding of the relationship between specific apple genes and apple root resistance traits is critical for future development and deployment of molecular tools for efficient and precise incorporation of resistance traits into the next generation of resistant apple rootstocks.

## References

[1] Janick J, Cummins J, Brown S, Hemmat M (1996). Apples. Fruit. Breed..

[2] Jaffee B, Abawi G, Mai W (1982). Fungi associated with roots of apple seedlings grown in soil from an apple replant site. Plant Dis..

[3] Mazzola M (1998). Elucidation of the microbial complex having a causal role in the development of apple replant disease in Washington. Phytopathology.

[4] Mazzola M (1997). Identification and pathogenicity of *Rhizoctonia* spp. isolated from apple roots and orchard soils. Phytopathology.

[5] Tewoldemedhin YT, Mazzola M, Botha WJ, Spies CF, McLeod A (2011). Characterization of fungi (*Fusarium* and *Rhizoctonia*) and oomycetes (*Phytophthora* and *Pythium*) associated with apple orchards in South Africa. Eur. J. Plant Pathol..

[6] Okubara PA, Dickman MB, Blechl AE (2014). Molecular and genetic aspects of controlling the soilborne necrotrophic pathogens *Rhizoctonia* and *Pythium*. Plant Sci..

[7] Zhu Y, Fazio G, Mazzola M (2014). Elucidating the molecular responses of apple rootstock resistant to ARD pathogens: challenges and opportunities for development of genomics-assisted breeding tools. Hort. Res..

[8] Covey RP, Benson NR, Haglund WA (1979). Effect of soil fumigation on the apple replant disease in Washington. Phytopathology.

[9] Mazzola, M. & Strauss, S. Resilience of orchard replant soils to pathogen re-infestation in response to Brassicaceae seed meal amendment. *Asp. Appl. Biol*. **119**, 69–77 (2013).

[10] Cummins JN, Aldwinckle HS (1995). Breeding rootstocks for tree fruit crops. N. Z. J. Crop Hortic. Sci..

[11] Fazio G, Robinson TL, Aldwinckle HS (2015). The Geneva apple rootstock breeding program. Plant Breed. Rev..

[12] Collard BC, Mackill DJ (2008). Marker-assisted selection: an approach for precision plant breeding in the twenty-first century. Philos. Trans. R. Soc. Lond. B Biol. Sci..

[13] Fiorani F, Schurr U (2013). Future scenarios for plant phenotyping. Annu. Rev. Plant Biol..

[14] Ogura T, Busch W (2016). Genotypes, networks, phenotypes: moving toward plant systems genetics. Annu. Rev. Cell. Dev. Biol..

[15] Bazakos C, Hanemian M, Trontin C, Jiménez-Gómez JM, Loudet O (2017). New strategies and tools in quantitative genetics: how to go from the phenotype to the genotype. Annu. Rev. Plant Biol..

[16] Huijser P, Schmid M (2011). The control of developmental phase transitions in plants. Development.

[17] Hsu PY, Harmer SL (2014). Wheels within wheels: the plant circadian system. Trends Plant Sci..

[18] Tsuda K, Somssich IE (2015). Transcriptional networks in plant immunity. N. Phytol..

[19] Dodds PN, Rathjen JP (2010). Plant immunity: towards an integrated view of plant-pathogen interactions. Nat. Rev. Genet..

[20] Dangl JL, Horvath DM, Staskawicz BJ (2013). Pivoting the plant immune system from dissection to deployment. Science.

[21] Erktan A, McCormack ML, Roumet C (2018). Frontiers in root ecology: recent advances and future challenges. Plant Soil.

[22] Delory, B. M., Weidlich, E. W. A., van Duijnen, R., Pagès, L. & Temperton, V. M. in *Methods**Mol. Biol.* **1761**, 3–22 (Humana Press Inc., 2018). 10.1007/978-1-4939-7747-5_1 29525945

[23] McCully ME (1999). Roots in soil: unearthing the complexities of roots and their rhizospheres. Annu. Rev. Plant Biol..

[24] De Coninck B, Timmermans P, Vos C, Cammue BPA, Kazan K (2015). What lies beneath: belowground defense strategies in plants. Trends Plant Sci..

[25] Velasco R (2010). The genome of the domesticated apple (*Malus*× *domestica* Borkh.). Nat. Genet..

[26] Pritchard L, Birch PR (2014). The zigzag model of plant–microbe interactions: is it time to move on?. Mol. Plant Pathol..

[27] Cui H, Tsuda K, Parker JE (2015). Effector-triggered immunity: from pathogen perception to robust defense. Annu. Rev. Plant Biol..

[28] Jones JD, Dangl JL (2006). The plant immune system. Nature.

[29] Boller T, Felix G (2009). A renaissance of elicitors: perception of microbe-associated molecular patterns and danger signals by pattern-recognition receptors. Annu. Rev. Plant Biol..

[30] Bonardi V, Dangl JL (2012). How complex are intracellular immune receptor signaling complexes?. Front. Plant Sci..

[31] Chisholm ST, Coaker G, Day B, Staskawicz BJ (2006). Host-microbe interactions: shaping the evolution of the plant immune response. Cell.

[32] Boller T, He SY (2009). Innate immunity in plants: an arms race between pattern recognition receptors in plants and effectors in microbial pathogens. Science.

[33] Asai S, Shirasu K (2015). Plant cells under siege: plant immune system versus pathogen effectors. Curr. Opin. Plant Biol..

[34] Glazebrook J (2005). Contrasting mechanisms of defense against biotrophic and necrotrophic pathogens. Annu. Rev. Phytopathol..

[35] Bari R, Jones JD (2009). Role of plant hormones in plant defence responses. Plant Mol. Biol..

[36] Mengiste T (2012). Plant immunity to necrotrophs. Annu. Rev. Phytopathol..

[37] Robert-Seilaniantz A, Grant M, Jones JD (2011). Hormone crosstalk in plant disease and defense: more than just jasmonate-salicylate antagonism. Annu. Rev. Phytopathol..

[38] Verhage A, van Wees SC, Pieterse CM (2010). Plant immunity: it’s the hormones talking, but what do they say?. Plant Physiol..

[39] Grant MR, Jones JD (2009). Hormone (dis) harmony moulds plant health and disease. Science.

[40] Moore JW, Loake GJ, Spoel SH (2011). Transcription dynamics in plant immunity. Plant Cell.

[41] Beckers G, Spoel S (2006). Fine-tuning plant defence signalling: salicylate versus jasmonate. Plant Biol..

[42] Pieterse CM, Leon-Reyes A, Van der Ent S, Van Wees SC (2009). Networking by small-molecule hormones in plant immunity. Nat. Chem. Biol..

[43] Birkenbihl RP, Somssich IE (2011). Transcriptional plant responses critical for resistance towards necrotrophic pathogens. Front. Plant Sci..

[44] Hammerschmidt R (1999). Phytoalexins: what have we learned after 60 years?. Annu. Rev. Phytopathol..

[45] VanEtten HD, Mansfield JW, Bailey JA, Farmer EE (1994). Two classes of plant antibiotics: phytoalexins versus” phytoanticipins”. Plant Cell.

[46] Dixon RA (2001). Natural products and plant disease resistance. Nature.

[47] Zhao N, Wang G, Norris A, Chen X, Chen F (2013). Studying plant secondary metabolism in the age of genomics. Crit. Rev. Plant Sci..

[48] Grayer RJ, Kokubun T (2001). Plant–fungal interactions: the search for phytoalexins and other antifungal compounds from higher plants. Phytochemistry.

[49] Li Y (2014). Multiple rice microRNAs are involved in immunity against the blast fungus *Magnaporthe oryzae*. Plant Physiol..

[50] Weiberg A, Jin H (2015). Small RNAs—the secret agents in the plant–pathogen interactions. Curr. Opin. Plant Biol..

[51] Fei Q, Zhang Y, Xia R, Meyers BC (2016). Small RNAs add zing to the zig-zag-zig model of plant defenses. Mol. Plant Microbe Interact..

[52] Zhu Y, Shao J, Zhou Z, Davis RE (2019). Genotype-specific suppression of multiple defense pathways in apple root during infection by *Pythium ultimum*. Hort. Res..

[53] Zhu, Y., Zhao, J. & Zhou, Z. Identifying an elite panel of apple rootstock germplasm with contrasting root resistance to *Pythium ultimum*. *J. Plant Pathol. Microbiol*. **9**10.4172/2157-7471.1000461 (2018).

[54] Zhou Z, Tian Y, Cong P, Zhu Y (2018). Functional characterization of an apple *(Malus* x *domestica*) LysM domain receptor encoding gene for its role in defense response. Plant Sci..

[55] Zhu Y, Shao J, Zhou Z, Davis RE (2017). Comparative transcriptome analysis reveals a preformed defense system in apple root of a resistant genotype of G.935 in the absence of pathogen. Int. J. Plant Genomics.

[56] Shin S (2016). Transcriptome changes specifically associated with apple *(Malus domestica*) root defense response during *Pythium ultimum* infection. Physiol. Mol. Plant Pathol..

[57] Atucha A, Emmett B, Bauerle TL (2014). Growth rate of fine root systems influences rootstock tolerance to replant disease. Plant Soil.

[58] Emmett B, Nelson EB, Kessler A, Bauerle TL (2014). Fine-root system development and susceptibility to pathogen colonization. Planta.

[59] Lucas M, Balbín-Suárez A, Smalla K, Vetterlein D (2018). Root growth, function and rhizosphere microbiome analyses show local rather than systemic effects in apple plant response to replant disease soil. PLoS ONE.

[60] Benfey, P. N. Toward a systems analysis of the root. In *Cold Spring Harbor Symp. Quant. Biol*. **77**, 91–96 (2012). 10.1101/sqb.2012.77.014506 PMC3883509 23234807

[61] Fazio, G., Robinson, T. L. & Aldwinckle, H. S. The Genava apple rootstock breeding program. In *Plant Breed. Rev*. **39**, 379–424 (Wiley-Blackwell, 2015).

[62] Russo NL, Robinson TL, Fazio G, Aldwinckle HS (2007). Field evaluation of 64 apple rootstocks for orchard performance and fire blight resistance. HortScience.

[63] Weiß S, Liu B, Reckwell D, Beerhues L, Winkelmann T (2017). Impaired defense reactions in apple replant disease-affected roots of *Malus domestica* ‘M26’. Tree Physiol..

[64] Weiss S, Bartsch M, Winkelmann T (2017). Transcriptomic analysis of molecular responses in *Malus domestica* ‘M26’ roots affected by apple replant disease. Plant Mol. Biol..

[65] Reim, S. et al. Evaluation of *Malus* genetic resources for tolerance to apple replant disease (ARD). *Sci. Hort*. **256**, 108517 (2019).

[66] Abdollahi H, Rugini E, Ruzzi M, Muleo R (2004). In vitro system for studying the interaction between *Erwinia amylovora* and genotypes of pear. Plant Cell Tiss. Org. Cult..

[67] Bhatti S, Jha G (2010). Current trends and future prospects of biotechnological interventions through tissue culture in apple. Plant Cell Rep..

[68] Zhu Y, Saltzgiver M, Zhao J (2018). A phenotyping protocol for detailed evaluation of apple root resistance responses utilizing tissue culture micropropagated apple plants. Am. J. Plant Sci..

[69] Zhu Y, Shin S, Mazzola M (2016). Genotype responses of two apple rootstocks to infection by *Pythium ultimum* causing apple replant disease. Can. J. Plant Pathol..

[70] Winkelmann T (2019). Apple replant disease: causes and mitigation strategies. Curr. Issues Mol. Biol..

[71] Mazzola M, Manici LM (2012). Apple replant disease: role of microbial ecology in cause and control. Annu. Rev. Phytopathol..

[72] Metzker ML (2010). Sequencing technologies—the next generation. Nat. Rev. Genet..

[73] van Hulten M, Pelser M, Van Loon L, Pieterse CM, Ton J (2006). Costs and benefits of priming for defense in Arabidopsis. Proc. Natl Acad. Sci. USA.

[74] Zhou Z, Cong P, Tian Y, Zhu Y (2017). Using RNA-seq data to select reference genes for normalizing gene expression in apple roots. PLoS ONE.

[75] Katiyar-Agarwal S, Jin H (2010). Role of small RNAs in host-microbe interactions. Annu. Rev. Phytopathol..

[76] Chaloner T, van Kan JA, Grant-Downton RT (2016). RNA ‘Information Warfare’in pathogenic and mutualistic interactions. Trends Plant Sci..

[77] Daccord N (2017). High-quality de novo assembly of the apple genome and methylome dynamics of early fruit development. Nat. Genet..

[78] Altpeter F (2016). Advancing crop transformation in the era of genome editing. Plant Cell.

[79] Schiml S, Puchta H (2016). Revolutionizing plant biology: multiple ways of genome engineering by CRISPR/Cas. Plant Methods.

[80] Belhaj K, Chaparro-Garcia A, Kamoun S, Nekrasov V (2013). Plant genome editing made easy: targeted mutagenesis in model and crop plants using the CRISPR/Cas system. Plant Methods.

[81] Belhaj K, Chaparro-Garcia A, Kamoun S, Patron NJ, Nekrasov V (2015). Editing plant genomes with CRISPR/Cas9. Curr. Opin. Biotechnol..

[82] Lloyd SR, Schoonbeek H-j, Trick M, Zipfel C, Ridout CJ (2014). Methods to study PAMP-triggered immunity in *Brassica* species. Mol. Plant Microbe Interact..

[83] Taheri P, Kakooee T (2017). Reactive oxygen species accumulation and homeostasis are involved in plant immunity to an opportunistic fungal pathogen. J. Plant Physiol..

[84] Luna E (2011). Callose deposition: a multifaceted plant defense response. Mol. Plant Microbe Interact..

[85] Flors V, Ton J, Jakab G, Mauch-Mani B (2005). Abscisic acid and callose: team players in defence against pathogens?. J. Phytopathol..

[86] Denness L (2011). Cell wall damage-induced lignin biosynthesis is regulated by a reactive oxygen species- and jasmonic acid-dependent process in Arabidopsis. Plant Physiol..

[87] Cheng, S. et al. Characterization and expression patterns of a cinnamate-4-hydroxylase gene involved in lignin biosynthesis and in response to various stresses and hormonal treatments in Ginkgo biloba. *Acta Physiol. Plant*10.1007/s11738-017-2585-4 (2018).

[88] Ton J, Mauch‐Mani B (2004). *β*-amino-butyric acid-induced resistance against necrotrophic pathogens is based on ABA-dependent priming for callose. Plant J..

[89] Jin, L. & Mackey, D. M. Measuring callose deposition, an indicator of cell wall reinforcementin. In *Plant Pattern Recognition Receptors* 195–205 (Springer, 2017). 10.1007/978-1-4939-6859-6_16 28220426

[90] Wang JW, Wu JY (2005). Nitric oxide is involved in methyl jasmonate-induced defense responses and secondary metabolism activities of Taxus cells. Plant Cell Physiol..

[91] Cobb JN, DeClerck G, Greenberg A, Clark R, McCouch S (2013). Next-generation phenotyping: requirements and strategies for enhancing our understanding of genotype–phenotype relationships and its relevance to crop improvement. Theor. Appl. Genet..

[92] Weiß S, Winkelmann T (2017). Transcriptome profiling in leaves representing aboveground parts of apple replant disease affected *Malus domestica* ‘M26’ plants. Sci. Hort..

